# Supplementary material for: Topological network properties of resting-state functional connectivity patterns are associated with metal mixture exposure in adolescents
Source: Front Neurosci. 2023 Feb 6;17:1098441. doi: 10.3389/fnins.2023.1098441 (PMC9939635; doi:10.3389/fnins.2023.1098441)
Supplement: Supplementary file 1 [file Data_Sheet_1.docx]

# **Supplementary Materials**

**
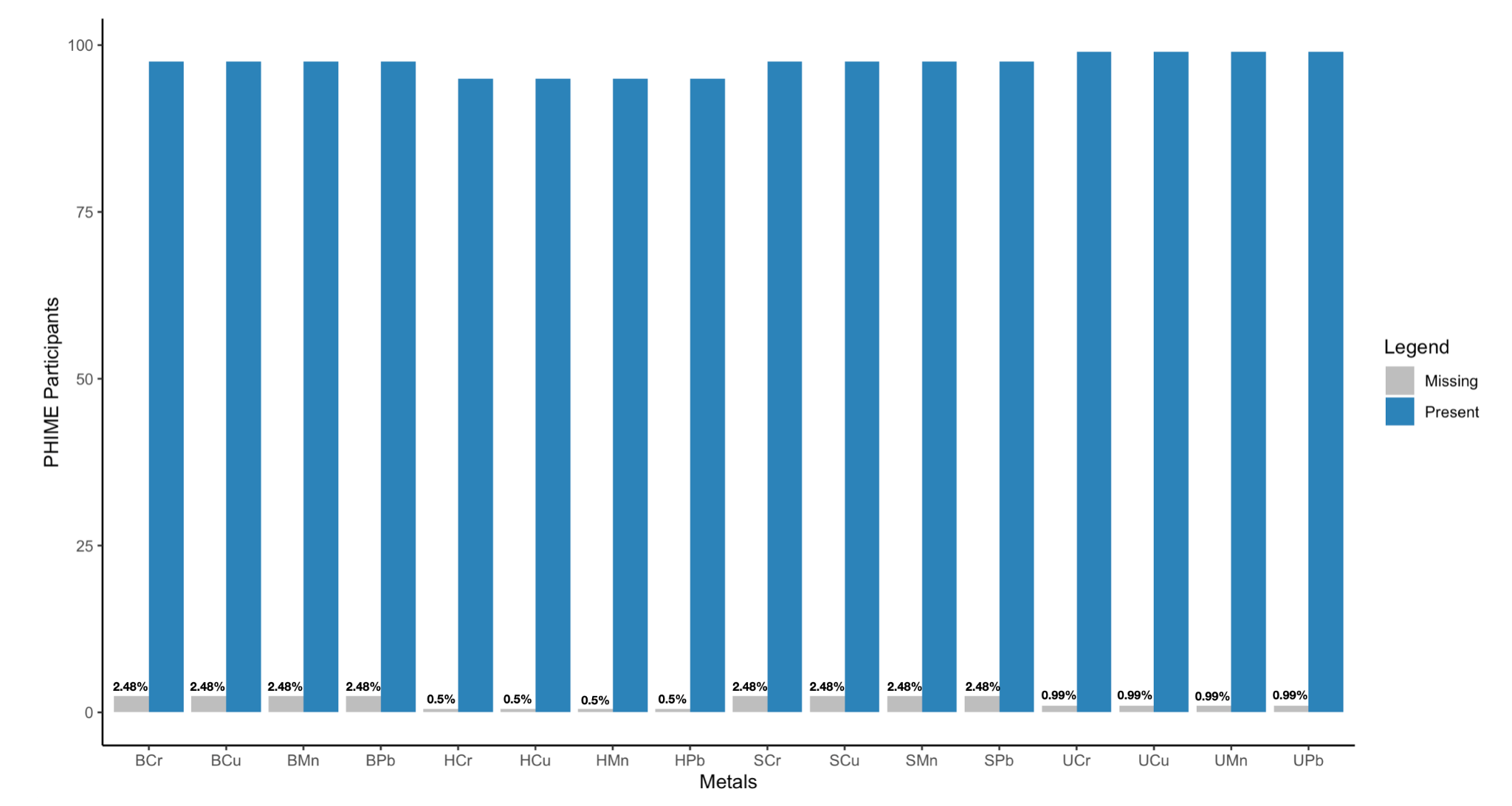
**

**Figure S1 - PHIME-MRI biomarker.** Complete overview of all biomarkers collected in PHIME-MRI. The gray bars represent the missing values while the blue ones the collected and analyzed values for each PHIME-MRI participant. Percentage of missing data for each component is reported on the top of each gray bar. Components abbreviations: the first letter represents the medium (S=saliva, B=blood, U=urine, H=hair) and the second and third letters represent the metals (Mn=manganese, Pb=lead, Cr=chromium, Cu=copper).


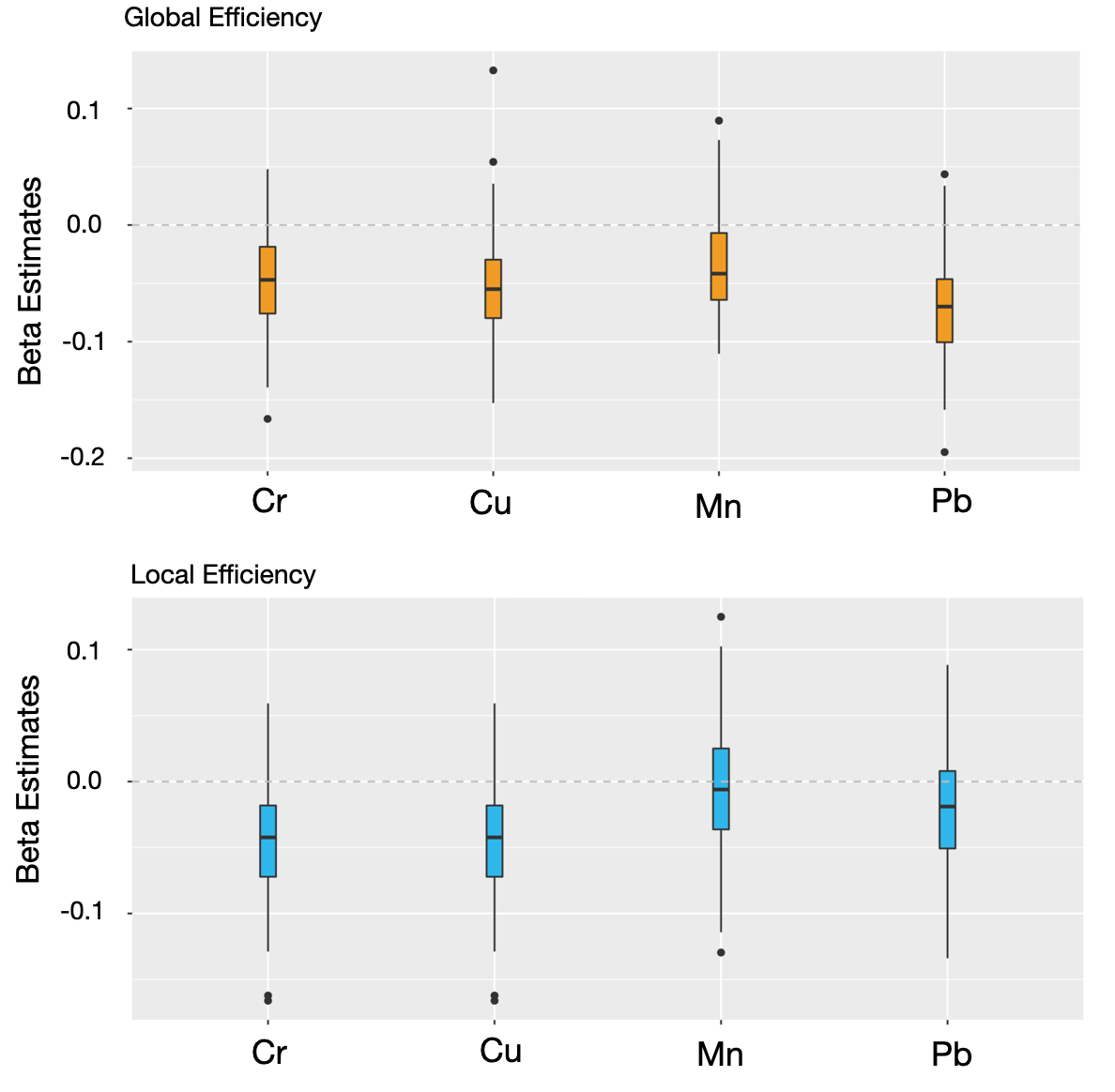


**Figure S2 - MMBs approach: first level.** Betas and 95% confidence interval obtained from the WQS association between each metal and GE (orange bar) or LE (blue bar) was estimated among 192 adolescents included in the current study. All models were adjusted for sex and age. Components abbreviations represent the metals (Mn=manganese, Pb=lead, Cr=chromium, Cu=copper).


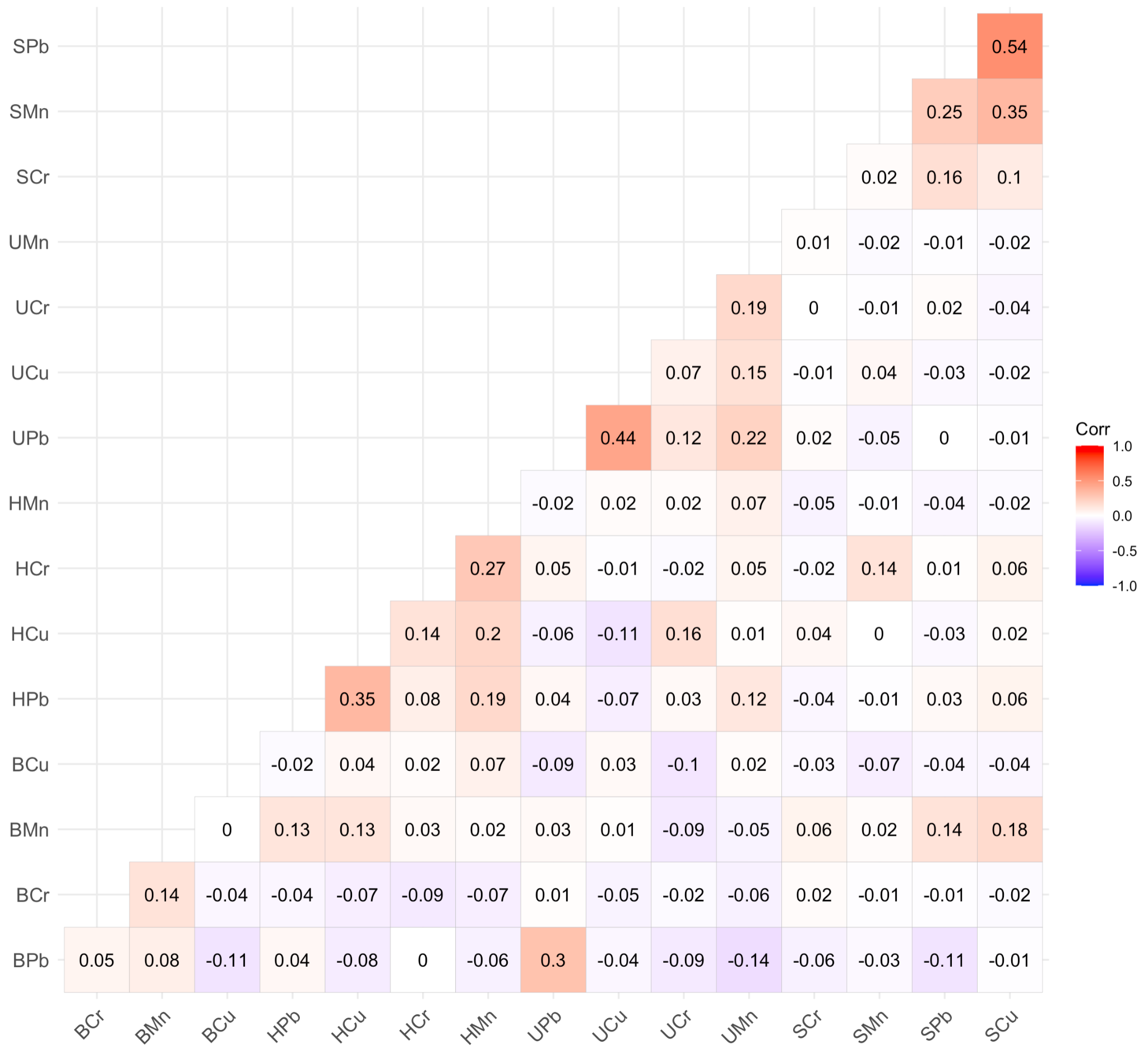


**Figure S3 - Heatmap of metals exposure.** Pearson’s correlations between all biomarkers collected in PHIME-MRI. Components abbreviations: the first letter represents the medium (S=saliva, B=blood, U=urine, H=hair) and the second and third letters represent the metals (Mn=manganese, Pb=lead, Cr=chromium, Cu=copper).
